# Novel diagnostic biomarkers of oxidative stress, ferroptosis, immune infiltration characteristics and experimental validation in ischemic stroke

**DOI:** 10.18632/aging.205415

**Published:** 2024-01-09

**Authors:** Kaisheng Yuan, Xiao Jin, Xiaocong Mo, Ruiqi Zeng, Xu Zhang, Qiufang Chen, Ling Jin

**Affiliations:** 1 Department of Metabolic and Bariatric Surgery, The First Affiliated Hospital of Jinan University, Jinan University, Guangzhou, China; 2 Department of Traditional Chinese Medicine, The First Affiliated Hospital of Jinan University, Jinan University, Guangzhou, China; 3 Department of Oncology, The First Affiliated Hospital of Jinan University, Jinan University, Guangzhou, China; 4 Department of Urology, The Second Peoples Hospital of Yibin City, Yibin, China; 5 Department of Basic Medicine, Harbin Medical University, Harbin, China

**Keywords:** ischemic stroke, oxidative stress, ferroptosis, immune infiltration, PTGS2

## Abstract

Ischemic stroke (IS) is a prominent type of cerebrovascular disease leading to death and disability in an aging society and is closely related to oxidative stress. Gene expression profiling (GSE222551) was derived from Gene Expression Omnibus (GEO), and 1934 oxidative stress (OS) genes were obtained from the GeneCards database. Subsequently, we identified 149 differentially expressed genes related to OS (DEOSGs). Finally, PTGS2, FOS, and RYR1 were identified as diagnostic markers of IS. Moreover, GSE16561 was used to validate the DEOSGs. Two diagnostic genes (PTGS2 and FOS) were significantly highly expressed, while RYR1 was significantly lowly expressed in the IS group. Remarkably, immune infiltration characteristics of these three genes were analyzed, and we found that PTGS2, FOS, and RYR1 were mainly correlated with Mast cells activated, Neutrophils, and Plasma cells, respectively. Next, we intersected three DEOSGs with the ferroptosis gene set, the findings revealed that only PTGS2 was a differentially expressed gene of ferroptosis. High PTGS2 expression levels in the infarcted cortex of middle cerebral artery occlusion (MCAO) rats were confirmed by immunofluorescence (IF), western blotting (WB), and Immunohistochemistry (IHC). Inhibition of PTGS2 clearly improved the neurological outcome of rats by decreasing infarct volume, neurological problems, and modified neurological severity scores following IS compared with the controls. The protective effect of silencing PTGS2 may be related to anti-oxidative stress and ferroptosis. In conclusion, this work may provide a new perspective for the research of IS, and further research based on PTGS2 may be a breakthrough.

## INTRODUCTION

Ischemic stroke (IS) remains one of the leading causes of death and long-term disability in patients worldwide, which brings tremendous treatment difficulties and financial burdens on current healthcare systems [1–3]. Despite the development of many early diagnostic tools and treatment methods to improve neurological outcomes for IS in recent years, the treatment outcome of patients is still unstable.

The occurrence of IS can induce a battery of biochemical or cellular responses, among which excessive reactive oxygen species (ROS) are typically performance [4–6]. Studies have demonstrated that oxidative stress (OS) will occur when the inherent antioxidant system cannot fully neutralize ROS and continues to maintain the endogenous redox balance. Once OS occurs, ROS can lead to massive cytotoxicity through oxidative damage of lipids and nucleic acids, ultimately causing scathing consequences to important structures and functions of brain tissue [7–10]. Excessive generation of reactive oxygen species (ROS) in the brain, triggers an overwhelming burden of ROS. This, in turn, leads to apoptosis in endothelial cells, modifications in the expression and/or assembly of occludin, ZO-1, and claudin-5, resulting in increased permeability of the blood-brain barrier (BBB) [11–13].

In our current work, we identified the molecular markers of OS, and revealed their subtle physiological functions using the machine learning algorithm. Moreover, with the experimental validation, inhibition of PTGS2 may alleviate the cerebral ischemic damage partly by anti-oxidative stress and ferroptosis. PTGS2, also known as prostaglandin endoperoxide synthase 2, is a key rate-limiting enzyme in prostaglandin biosynthesis [14]. Previous studies have indicated the involvement of PTGS2 in the regulation of angiogenesis in various cancer and tumor-related diseases. Furthermore, abnormal upregulation of PTGS2 has been observed in patients with IS [15]. Our findings would provide a deeper valuable reference for predicting biomarkers and the diagnosis regimens of IS.

## METHODS

### Data collection and processing

**Figure 1:**
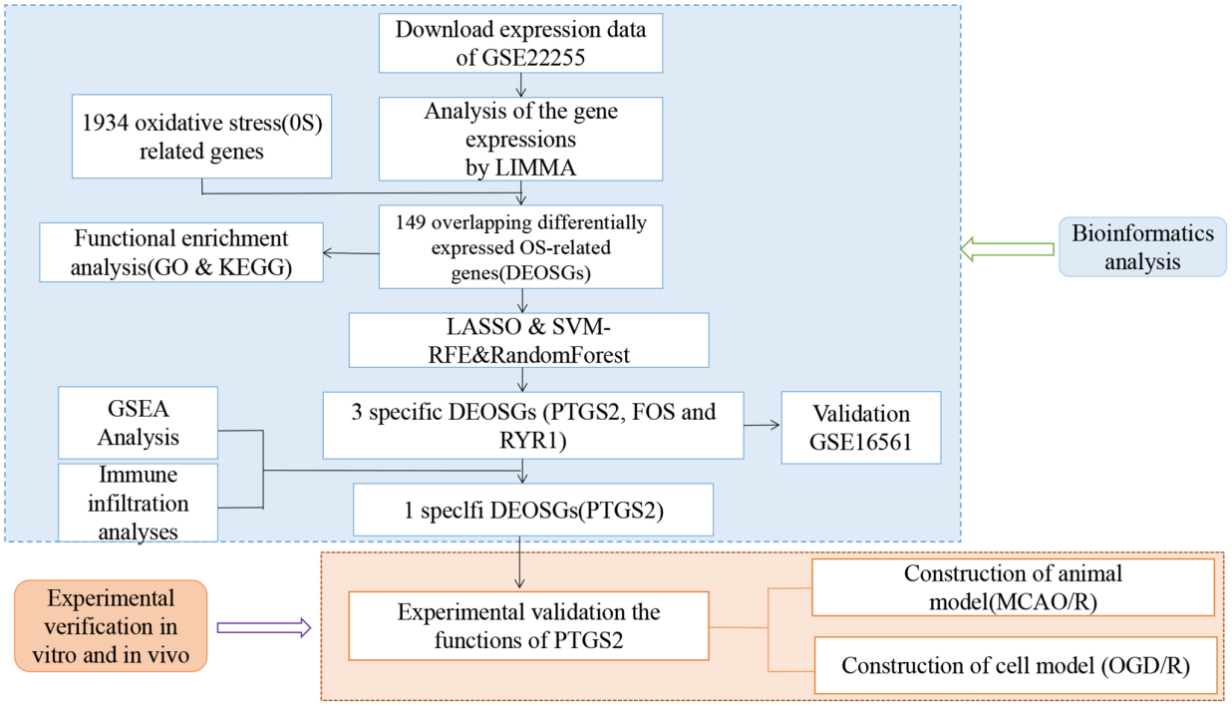
Flow chart of the study.

Gene expression profiling in this work (GSE22255; GSE16561) was downloaded from the NCBI-GEO database (http://www.ncbi.nlm.nih.gov/geo). GSE22255 included 20 IS patients and 20 age- and sex-matched controls using the GPL570 platform. The age range of the patients included was ≥18 and ≤75 years old [16]. Controls had no family history of stroke and were genetically unrelated to the included IS patients. GSE16561 included 39 IS patients and 24 controls using the GPL6883 platform. The patients’ age ranged from ≥18 to ≤90 years old [17]. Patients were diagnosed with IS on MRI, and controls were non-stroke neurologically healthy. The files were quantile-normalized, and transformed after merging. The analysis of differentially expressed genes was executed by the “limma” package with the criteria of the adjusted *P*-value <0.05. The GeneCards database (https://www.genecards.org/) was searched using the keyword “oxidative stress” and retrieved 10,022 OS related gene symbols. Then, we set the relevance score ≥4.0 as the cutoff value and finally obtained 1934 OS-related genes (Supplementary Table 1). Finally, we identified 149 differentially expressed genes of oxidative stress (DEOSGs) via the Venn diagram online tool. The workflow chart is shown in Figure 1.

### KEGG and GO enrichment analyses

Gene Ontology (GO) and Kyoto Encyclopedia of Genes and Genomes (KEGG) pathway enrichment analyses [18] were conducted by R package “clusterProfiler” [19] for the target DEOSGs’ function annotating and pathway predicting. Only terms with adjusted. *P* < 0.05 was considered statistically significant.

### Validation of the specific DEOSGs in the IS

The least absolute shrinkage and selection operator (LASSO) logistic regression [20] with the “glmnet” package, RandomForest with “random Forest” package and the support vector machine-recursive feature elimination (SVM-RFE) [21] with the “e1071” package were applied to screen the specific DEOSGs. The data obtained by the three machine algorithms were analyzed and presented by Venn diagram. The intersection of DEOSGs owned highly important function and would be used in subsequent research.

### The ROC curve and expression analysis

In the GSE22255 dataset, we executed receiver operating characteristic (ROC) (“pROC” package) curve analysis on each specific DEOSGs to evaluate their accuracy. Expression levels of specific DEOSGs in GSE22255 were displayed in the color boxplots generated by the “ggplot2”. And GSE16561 was the verification group.

### GSEA analysis

The GSEA was applied to investigate the function of the diagnostic DEOSGs. *P* < 0.05 was used as the criterion for significant enrichment.

### Experimental animals and ethics statement

In this study, healthy adult male Sprague-Dawley (SD) rats weighing between 220–250 g were provided by Experimental Animal Center in Southern Medical University, Guangzhou, China. All experimental methods were conducted in agreement with the National Institutes of Health’s Guide for the Care and Use of Laboratory Animals and endorsed by the Institutional Animal Care and Use Committee of Jinan University. This study was approved by the Ethics committee of Jinan University.

### Construction of middle cerebral artery occlusion and reperfusion (MCAO/R) model

Transient or permanent middle cerebral artery occlusion (MCAO) is one of the closest models to simulate human ischemic stroke [22]. This model was selected for subsequent studies. In our study, after the rats were anesthetized, the common carotid artery and the external carotid artery were exposed and ligated. Nylon wires (0.24–0.28 mm in diameter) (Beijing Xinong Biotechnology, China) were inserted deeply into the brain middle artery of rats for 1 h after oblique incision away from the internal carotid artery. The surgical sutures were pulled to the site where the common carotid artery was ligated for the reperfusion of the middle cerebral artery after 1 h ischemia. Rats from the sham operation group (sham group) were not subjected the external carotid artery blockage, but the rest steps were the same.

### Cell culture and oxygen-glucose deprivation and reperfusion (OGD/R) treatment of primary neurons

Primary neuron cells were obtained from the brain cortex of newborn rats (24 hours old) and cultured in humidified incubator as previously depicted [23]. Approximately 2.0 × 10^6^ cells were seeded per well with 2 mL Neurobasal Medium including 2% B27 supplement + 1% streptomycin (100 U/mL) + 1% penicillin (100 U/mL). The neuron cells were cultured in 37°C humidified incubator containing 5% CO_2_ until 90% purity was achieved. OGD/R treatment was performed according to a method which was previously depicted [23]. In short, neuron cells were firstly washed and then cultured in glucose-free DMEM in humidified hypoxic incubator for 1.5 h. The hypoxic incubator is equilibrated with hypoxic gas (5% CO_2_ + 1% O_2_ + 94% N_2_) for 37°C. Then, the glucose-free DMEM was replaced by the Neurobasal Medium and cells were changed backed to the normoxia (nor.) incubator. The negative control was neuron cells which cultured in normoxia.

### ShRNA synthesis and injection

Sh-PTGS2 was synthesized by Cyagen Bioscience Inc (Guangzhou, China). The vector sh-NC was used as negative control. MCAO surgery was performed 21 days after interference. Sh-PTGS2 was injected into the left lateral ventricle of rats at the speed of 0.5 μl/min after animals were anesthetized with 10% chloral hydrate. Then, we fixed the injection needle for 10 min and slowly removed from the left lateral ventricle of rats within 5 min.

### Measurement of cerebral infarction volume

After 24 h reperfusion, the rats were deeply anesthetized and then decapitated. Their brain tissues were quickly removed and stored in −20°C for 20 min. Brains were sectioned into 3.0 mm-thick 5–6 coronal sections. The sections of brain were immersed in the 2% TTC solution (2,3,5-triphenyltetrazolium chloride, Sigma, USA) at 37°C for 40 min in a dark room. Subsequently, the slices were removed and fixed in 4% paraformaldehyde for 24 h. Then, the cerebral infraction volume was photographed with a digital camera and analyzed by ImageJ (ver1.37c, NIH) software.

### Evaluation of neurological deficits

Zea-Longa Neurological Deficit Score [24] was selected to measured neurofunctional outcomes of operated rats after MCAO/R 24 h. This score based on a 5-point scale, and rats within 1–3 points were selected as experimental rats, and the other have been rejected.

### RNA collection, reverse transcription, and RT-qPCR

RNA was collected from primary neuron cells by the use of TRIzol (Invitrogen, USA). The RNA concentration was the measured under Nanodrop ND-1000 spectrophotometry (Nanodrop Tech, USA) and RNA integrity was detected with denatured agarose gel electrophoresis. cDNA was acquired by reverse transcription using the SuperScript VILO cDNA Kit. The primers were constructed and synthesized by Sangon Biotechnology (Shanghai, China). RT-qPCR was conducted with the iQ5 RT-qPCR Detection System (Bio-Rad Laboratories, USA) following manufacturer’s instructions. Quantitative analysis of SLC7A11 and GPX4 was carried out using the SYBR Green Master Mix.

### Nissl staining

For Nissl staining, the treated sections were orderly stained by Nissl Stain Kit (Solarbio, Beijing, China), and washed by double distilled water for 5 min. After thoroughly transparentized by xylene for 3 min, the sheets were slowly sealed with neutral resin [25]. The ultrastructural pathological changes of each section were observed under optical microscope.

### Measurement of ROS, MDA, GSH and oxidative stress index (OSI)

Intracellular ROS level were detected by the ROS Assay Kit kits (Beyotime; S0033; China) according to the recommended manuals. The MDA and GSH levels were conducted using MDA Assay Kit (Beyotime; S0131; China) and GSH Assay Kit (Nanjing Jiancheng Bioengineering Institute; A006-2-1; China) according to the instructions. Moreover, oxidative stress was deeply measured by the oxidative stress index (OSI) which was the ratio of TOS (Randox Laboratories, UK): TAC (Randox Laboratories, UK) [26].

### Western blot

The brains were washed directly by saline perfusion and then the proteins of the brain tissues were cracked in RIPA buffer (RIPA: PMSF = 100:1). After measured all needed densities, the prepared proteins (20–30 μg/lane) were separated by SDS-PAGE (10%, 60 min) and then transferred onto pre-activated PVDF membranes. After blocking in skimmed milk (TBST: mike = 10:1) for 2 h at 37°C, the treated membranes were incubated with anti-PTGS2 (#12282; 1:600; CST; USA), SLC7A11 (ab216876; 1:600; Abcam; USA), GPX4 (#52455; 1:800; CST; USA) and β-actin (#4970; 1:4000; CST; USA) overnight at 4°C, using Antibody Diluent Solution (Life-iLab, Shanghai, China). Next day, following treated with corresponding secondary antibody, the finished membranes were visualized by ECL kit.

### Statistical analysis

Differences were assessed using One-way analysis of variance (ANOVA) and Student’s *t*-test. All analyses were conducted using SPSS 25.0 software and GraphPad Prism 8.0.1. Experiments were performed independently three times. *P* < 0.05 was considered statistically significant.

### Data availability statement

The datasets were downloaded from the GEO (https://www.ncbi.nlm.nih.gov/geo/) in this study.

## RESULTS

### Identification of DEGs and DEOSGs

**Figure 2:**
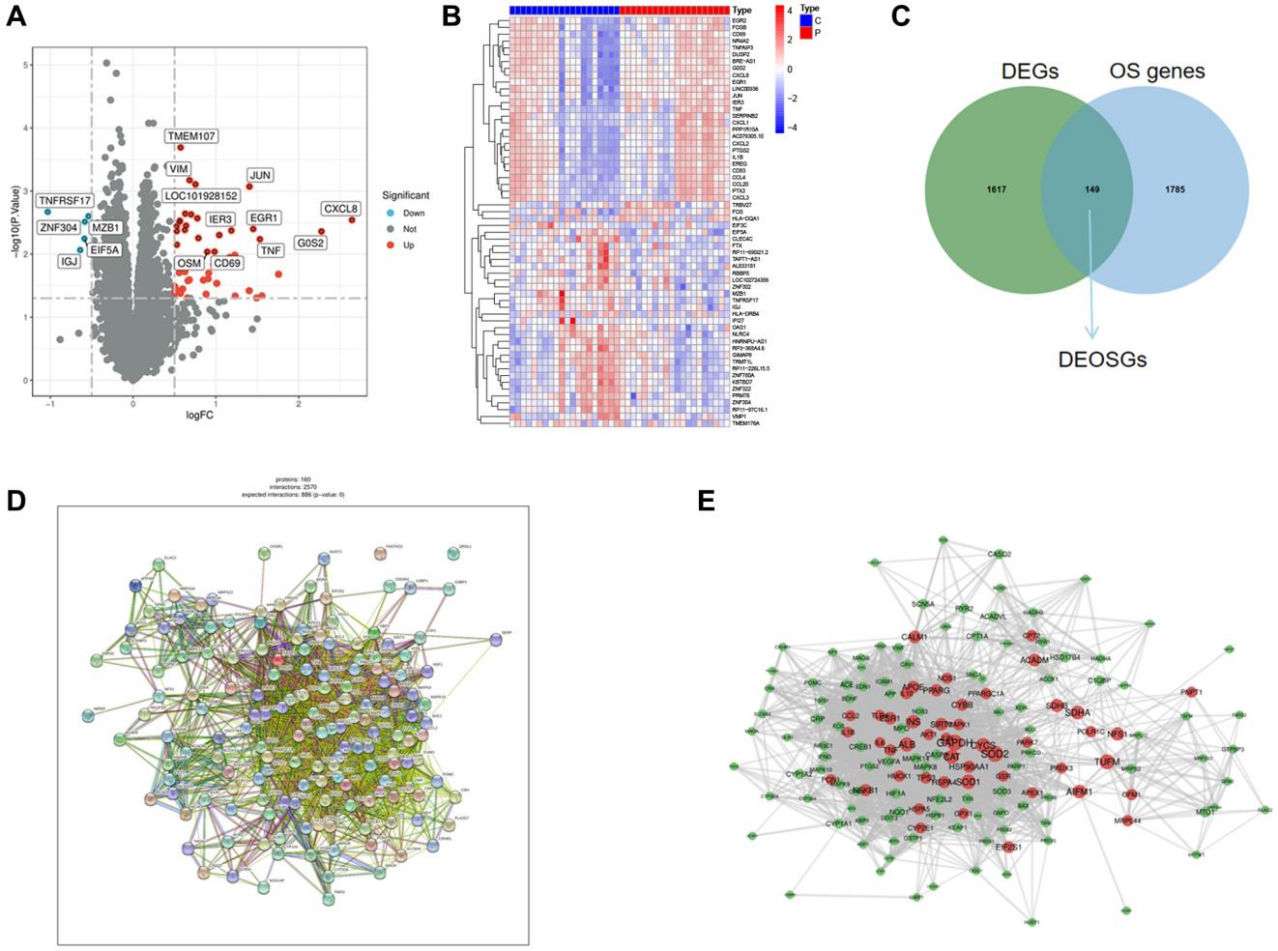
**Identification of DEGs and DEOSGs.** (**A**) Volcano plot of DEGs in GSE22255. (**B**) Heatmaps of DEGs in GSE22255. (**C**) Venn diagram shown the 149 overlaps of DEOSGs between DEGs in GSE22255 and OS genes. (**D**, **E**) PPI network of the 149 DEOSGs.

The analysis of differentially expressed genes (DGEs) (GSE22255) was performed by the “limma” package. A total of 1766 DEGs were obtained. Figure 2A, 2B showed the volcano and heatmap. Using the GeneCards (https://www.genecards.org/), a comprehensive database for searching and predicting a wide range of human genes, we obtained 1934 OS genes (relevance score ≥4) from GeneCards. Among the DEGs and OS genes, there were 149 overlapping items between DEGs and OS genes (Figure 2C). Here, we selected the 149 DEOSGs overlapping for subsequent researches. The PPI network of DEOSGs was visualized by Cytoscape software (Figure 2D, 2E).

### Biological functional enrichment research of DEOSGs

**Figure 3:**
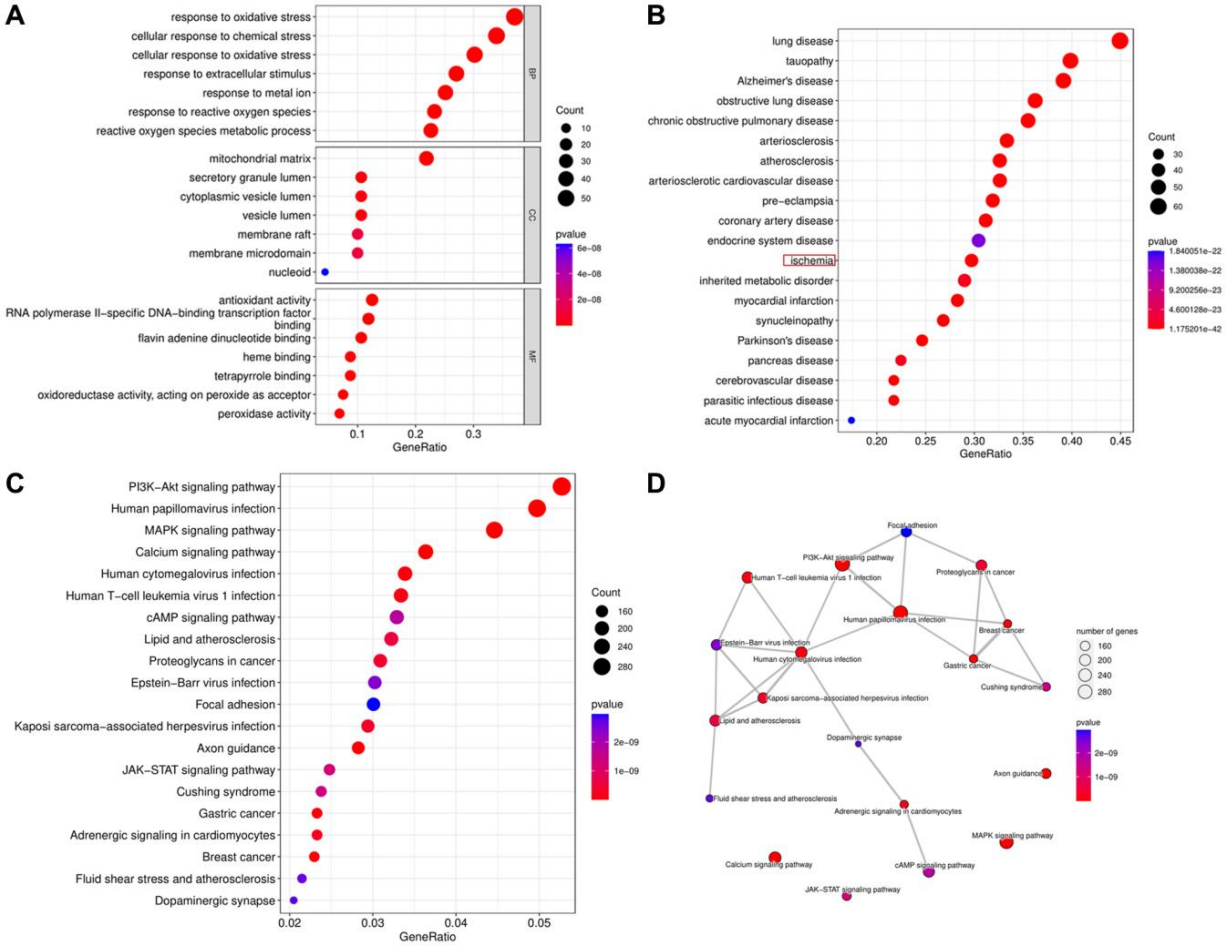
**Biological functional enrichment research of DEOSGs.** (**A**) GO analysis of DEOSGs. (**B**) DO analysis of DEOSGs. (**C**, **D**) KEGG analysis of DEOSGs.

We executed GO and KEGG databases to detect the potential and meaningful functions and related pathways of DEOSGs. The GO analysis clearly suggested that these DEOSGs in the biological processes (BP) were most enriched in “response to oxidative stress (GO:0006979)” and “cellular response to chemical stress (GO:0062197)”. These DEOSGs in cell component (CC) were most concentrated exist in “mitochondrial matrix (GO:0005759)” and “secretory granule lumen (GO:0034774)”. Alterations in molecular function (MF) were dominantly brimming with “antioxidant activity (GO:0016209)” and “RNA polymerase II-specific DNA-binding transcription factor binding (GO:0061629)” (Figure 3A). Among the significant DO enrichment (Figure 3B), the ischemic disease occupied a higher position. Most importantly, “PI3K-Akt signaling pathway (hsa04151)”, “Human papillomavirus infection (hsa05165)” and “MAPK signaling pathway (hsa04010)” were highlighted in the KEGG analysis (Figure 3C, 3D).

### Identification of the specific DEOSGs in IS

**Figure 4:**
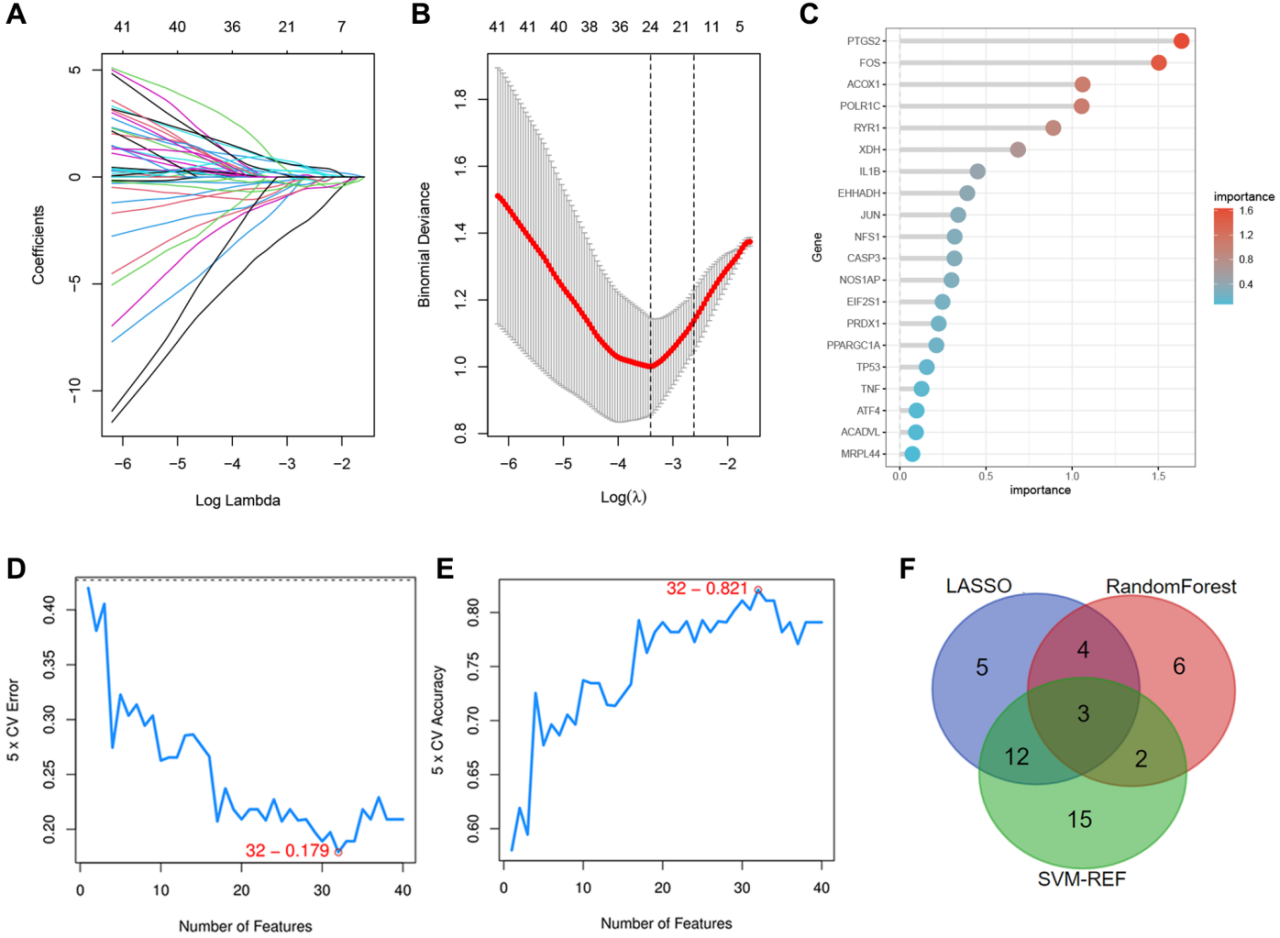
**Identification of the specific DEOSGs in IS.** (**A**, **B**) The LASSO logistic algorithm. (**C**) The important analysis of Random Forest algorithm. (**D**, **E**) The error and accuracy analysis of SVM-REF algorithm. (**F**) The intersection of the three algorithms (3 specific DEOSGs).

Next, we selected three novel machine learning algorithms to further screen out the key DEOSGs that are more important for the diagnosis and prognosis of our disease. We identified 24 specific DEOSGs (RYR1, FOS, PTGS2, PPARGC1A, NFS1, etc.,) with the LASSO logistic regression algorithm (Figure 4A, 4B). Furthermore, 15 specific DEOSGs (PTGS2, FOS, ACOX1, POLR1C, PTGS2, etc.,) were next filtered according to the Random Forest algorithm and the analysis of these 15 specific DEOSGs in Random Forest algorithm was shown in Figure 4C, where the more prominent the red color was, the more important the DEOSGs were for IS diagnosis. In addition, 32 specific DEOSGs (MPO, PRDX3, RYR1, STIP1, NOS1, etc.,) were then detected by SVM-REF algorithm, of which the error and accuracy analysis were showed in Figure 4D, 4E. Finally, 3 DEOSGs (PTGS2, FOS and RYR1) were selected by the combination of the three algorithms for the future validation (Figure 4F).

### Verification of the specific DEOSGs in IS

**Figure 5:**
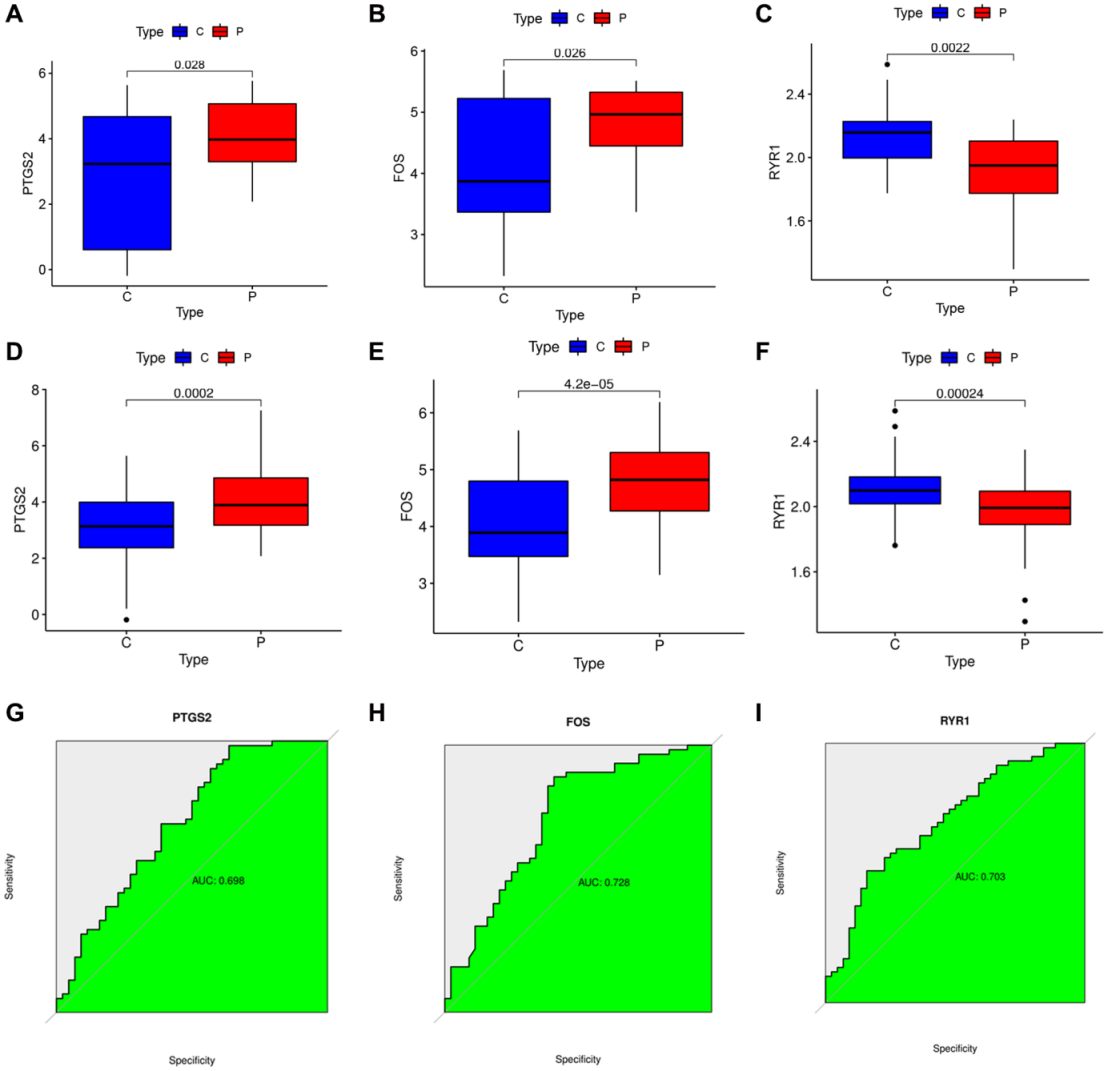
**Verification of the specific DEOSGs in IS.** (**A**) The expression of PTGS2 in GES22255 dataset. (**B**) The expression of FOS in GES22255 dataset. (**C**) The expression of RYR1 in GES22255 dataset. (**D**) The expression of PTGS2 was validated in the GSE16561. (**E**) The expression of FOS was validated in the GSE16561. (**F**) The expression of RYR1 was validated in the GSE16561. (**G**) The ROC curve of PTGS2. (**H**) The ROC curve of FOS. (**I**) The ROC curve of RYR1.

We explored the expressions of the 3 DEOSGs in the GSE22255 dataset and found that PTGS2 and FOS were highly expressed and RYR1 was lowly expressed in the IS group (Figure 5A–5C). And then GSE16561 was selected to further validate the expression value of these 3 DEOSGs. The trends of the two databases were consistent (Figure 5D–5F). Subsequently, we plotted receiver operating characteristic (ROC) curve to thoroughly test their efficacy. For PTGS2, the area under the curve (AUC) was 0.698 (Figure 5G). Notably, the AUC for FOS was 0.728 (Figure 5H), and the AUC for RYR1 was 0.703 (Figure 5I). All results indicated that the PTGS2, FOS and RYR1 had high diagnostic values in IS.

### GSEA analysis and immune cell infiltration

**Figure 6:**
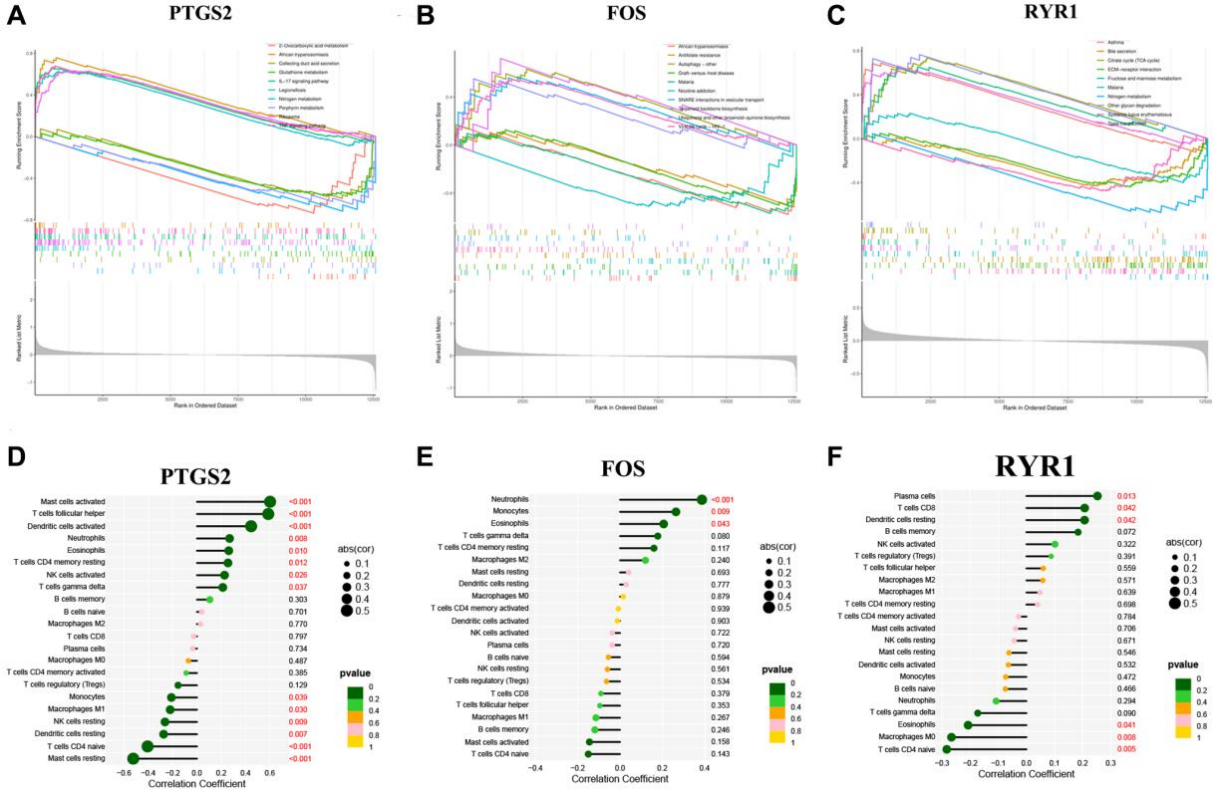
**GSEA analysis and immune cell infiltration.** (**A**) The GSEA analysis of PTGS2. (**B**) The GSEA analysis of FOS. (**C**) The GSEA analysis of RYR1. (**D**) The correlation analysis of immune cell infiltration with PTGS2. (**E**) The correlation analysis of immune cell infiltration with FOS. (**F**) The correlation analysis of immune cell infiltration with RYR1.

The potential functions of our 3 diagnostic DEOSGs were explored using GSEA. DEOSGs in the high-expression cohorts were highly enriched in inflammatory (such as NF-κB pathway), OS-related signaling pathway and atherosclerosis, respectively (Figure 6A–6C). After considering the founding of GSEA, we deeply explored the correlation between these three DEOSGs and inflammation. Figure 6D–6F confirmed the correlation between 3 diagnostic DEOSGs and 22 immune cells. Surprisingly, there existed obviously positive correlation between PTGS2 and 2 immune cells (Mast cells activated and T cells follicular helper), as well as obviously negative correlation between PTGS2 and Mast cells resting. Moreover, FOS was positively correlated with Neutrophils, but negatively correlated with T cells CD4 naive. Besides, RYR1 was positively correlated with Plasma cells, but negatively correlated with Macrophages M0 and T cells CD4 naive. These exploratory findings will guide us to further comprehend the critical role of these DEOSGs.

### The expression of PTGS2 after MCAO/R

**Figure 7:**
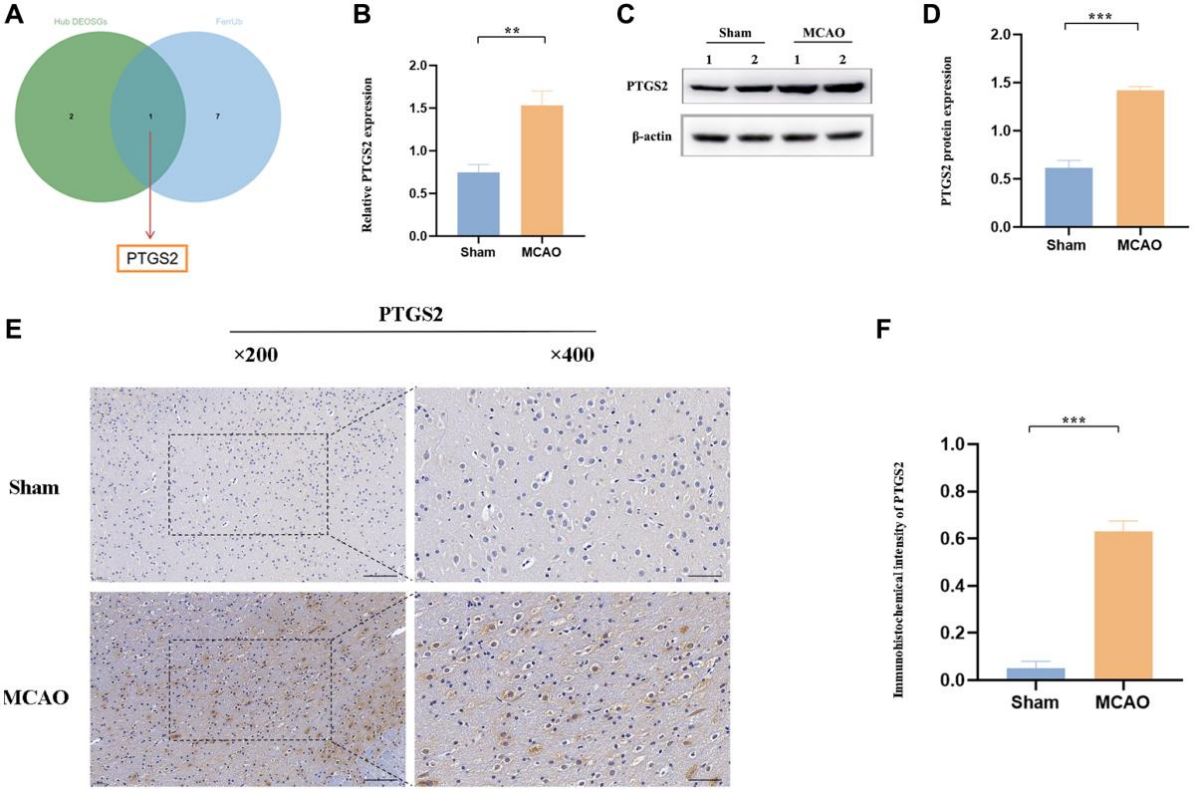
**The expression of PTGS2 after MCAO/R.** (**A**) Venn diagram showed that only PTGS2 was existed as upregulated ferroptosis-related DEGs. (**B**) RT-qPCR analysis of PTGS2 mRNA level. (**C**, **D**) Western blot analysis of PTGS2 protein level. (**E**, **F**) Immunohistochemical analysis of PTGS2 expression. ^*^p < 0.05; ^**^p < 0.01; ^***^p < 0.001.

Ferroptosis, a newly described non-apoptotic programmed pattern of iron-dependent cell death, also has been identified to cause substantial damage to the structural and functional integrity of brain tissue [27]. OS and ferroptosis both play fatal roles in the complicated pathological progress in IS, however, there are still few studies on the combination of them in IS. Here, among the specific DEOSGs, only PTGS2 was existed as upregulated ferroptosis-related DEGs (Figure 7A). Following our machine learning analysis described above, RT-qPCR, Western blot and IHC were conducted to clearly verify the role of PTGS2 in the ischemic cortex of rats. In Figure 7B–7D, not only mRNA level but also protein level showed the same significant increase (*P* < 0.05) trend of PTGS2 following MCAO/R 24 h compared with the control group. The results of IHC further confirmed the clear aggregation of PTGS2 in IS (Figure 7E, 7F).

### The effect of PTGS2 knockdown on neurological deficits and infarct volume after MCAO/R

**Figure 8:**
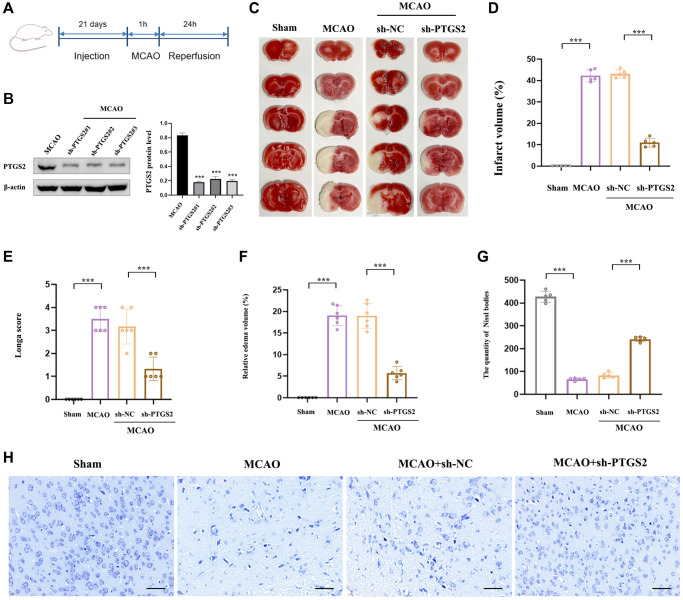
**Effects of PTGS2 knockdown on neurological deficits and infarct volume after MCAO/R.** (**A**) Experimental protocol schedule. (**B**) The efficiency of siRNA-mediated knockdown of PTGS2 in rats. (**C**, **D**) TTC staining. (**E**) Neurological score analysis. (**F**) Water content of the brain. (**G**, **H**) Nissl staining. ^*^p < 0.05; ^**^p < 0.01; ^***^p < 0.001.

In order to explore the specific role of PTGS2 in the pathological process of IS, we respectively injected sh-PTGS2 or sh-NC (negative control) into the left lateral ventricle of rats 21 days before surgery to induce the inhibition of PTGS2, and the schematic diagram of the experimental process was shown in Figure 8A. Figure 8B showed that the transfection was clearly successful in rats. TTC staining revealed that sh-PTGS2 administration could significantly reduce the volume of cerebral infarction compared with the MCAO group of rats (Figure 8C, 8D). Moreover, the neurological deficit scores and edema volume of rats were remarkably decreased following silencing PTGS2 (Figure 8E, 8F). More importantly, sh-PTGS2 rescued the neuronal damage, Nissl bodies decreased and nuclear shrinkage induced by MCAO (Figure 8G, 8H). These above evidences clearly indicated that silencing of PTGS2 improved the outcome of IS.

### Effects of PTGS2 knockdown on oxidative stress and ferroptosis of IS *in vitro*

**Figure 9:**
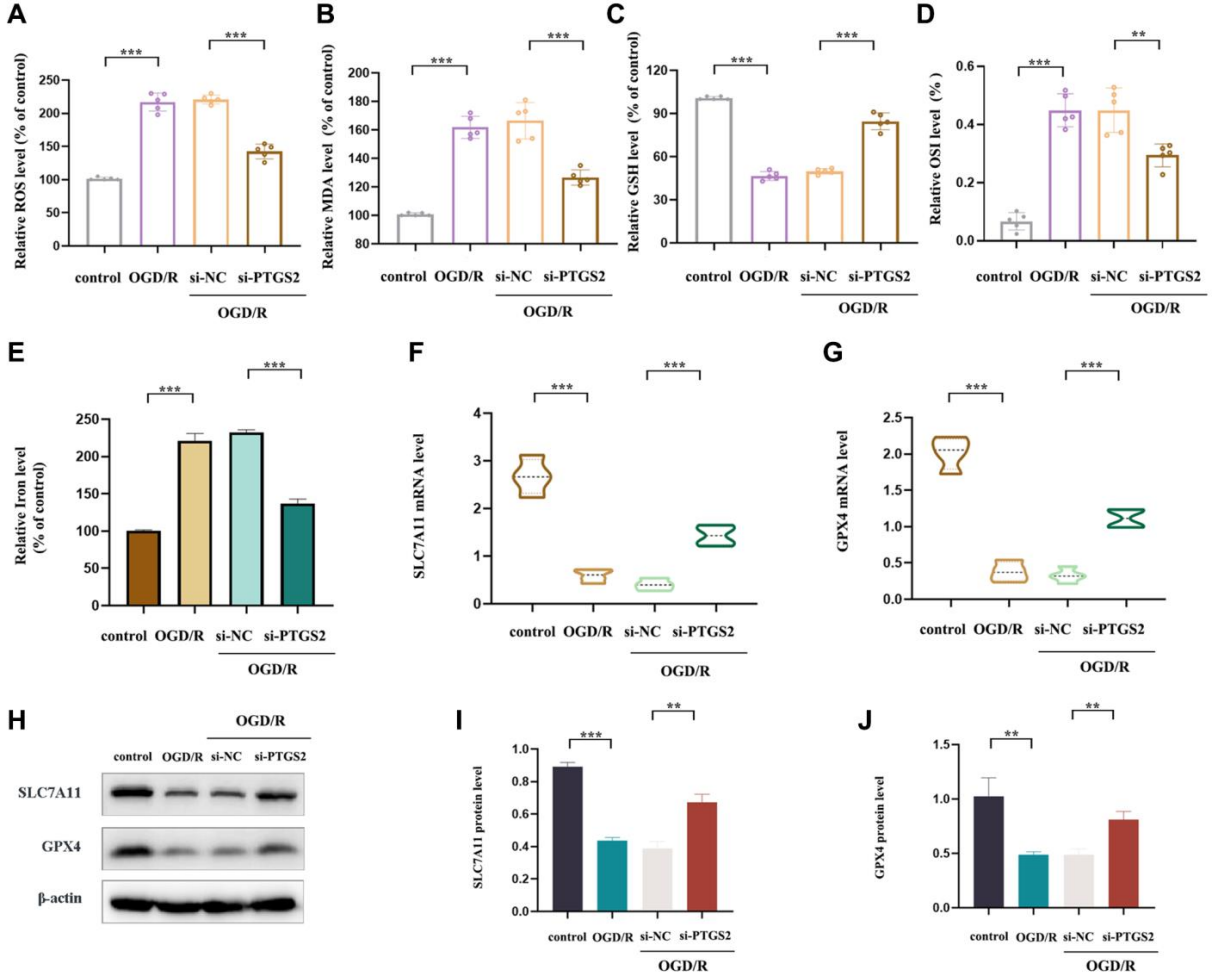
**Effects of PTGS2 knockdown on oxidative stress and ferroptosis of IS *in vitro*.** (**A**) ROS level of primary neuronal cells. (**B**) MDA level of primary neuronal cells. (**C**) GSH level of primary neuronal cells. (**D**) Oxidative stress index (OSI) level of primary neuronal cells. (**E**) Iron level of primary neuronal cells. (**F**, **G**) RT-qPCR analysis of SLC7A11 and GPX4 mRNA level. (**H**–**J**) Western blot analysis of SLC7A11 and GPX4 protein level. ^*^p < 0.05; ^**^p < 0.01; ^***^p < 0.001.

In order to further explore the specific protective mechanism of PTGS2 knockdown in the progression of IS, we detected the levels of MDA, ROS, GSH and OSI in primary neuronal cells post OGD/R (a model simulating brain ischemia *in vitro*). The results showed a significant increase in ROS, MDA and OSI levels, while an obviously decrease in GSH level, indicating that OGD/R was sufficient to cause OS injury in primary neuronal cells. Surprisingly, PTGS2 knockdown reversed this phenomenon (Figure 9A–9D). Ferroptosis is an iron-dependent programmed death triggered by lipid peroxide (LP) accumulation in the context of increased ROS production and GPX4 inactivation. PTGS2 has been found to be a positive regulator of ferroptosis [28]. Therefore, in order to detect whether the ameliorative effect of PTGS2 knockdown on IS was involved in ferroptosis, we further examined the characteristics of ferroptosis and the levels of key regulators iron, SLC7A11, and GPX4. As a result, it was consistent with expectations that the ischemic environment could remarkably increase iron content, while decreased the expression of SLC7A11 and GPX4 in primary neuronal cells, suggesting that OGD/R had ability to drive the occurrence of ferroptosis. In addition, PTGS2 knockdown rescued OGD/R-induced ferroptosis, mainly manifested in the reduction of iron content and the increase of SLC7A11 and GPX4 expression (Figure 9E–9J). To sum up, the protective effect of PTGS2 knockdown on IS may be partly related to its anti-oxidative stress and anti-ferroptosis.

## DISCUSSION

Ischemic stroke (IS), a common acute cerebrovascular disease, could cause massive neuronal death and severe neurological dysfunction [29]. Therefore, it has become the second leading cause of death worldwide. Studies have confirmed that after the IS, a large number of reactive oxygen species (ROS) occurred in nerve cells, leading to the accumulation of oxidative stress (OS) and brain injury [30, 31]. Thus, the prediction of biological targets based on OS may bring a new breakthrough for clinical treatment. However, few studies recently have focused their attention on unearthing biomarkers of abnormal expression involved in oxidative stress between IS and normal tissues.

In our current research, we identified 149 DEOSGs. The resulting GO enrichments revealed that these DEOSGs were primarily related to the oxidative stress and antioxidant activity. As we all know, although the pathogenesis of IS are complex, different evidences have successively demonstrated that the up-regulated mitogen-activated protein kinase (MAPK) in brain of ischemic patients release a key function in the activation of OS and inflammatory process [32, 33]. MAPKs participate in the regulation of flexile biomolecular processes [34] and are formed by three subfamilies, c-Jun N-terminal kinase (JNK) [35], p38 mitogen-activated protein kinase (p38) [35], and extracellular signal-regulated kinase (ERK)1/2. Of which, JNK is mainly stimulated under conditions of OS, inflammation and so on. In contrast, ERK 1/2 activation is strongly dependent on the release of growth factors and cytokines, as well as p38 is similarly activated by a mass of cytokines, stress, etc., [36]. Tian et al., has identified that inhibition of TXNIP could clearly alleviated OS Injury by triggering MAPK-Nrf2 axis in IS [32]. All these data consistently indicate that MAPK signaling plays a non-negligible role in the OS pathogenesis of IS and its related genes are also worthy of our attention. Surprisingly, here “MAPK signaling pathway (hsa04010)” was highlighted in the KEGG analysis.

PTGS2, FOS and RYR1 were identified as specific diagnostic DEOSG of IS using three novel machine learning algorithms (LASSO logistic regression, Random Forest and SVM-RFE). To be specific, we revealed that PTGS2 and FOS were significantly upregulated in IS (GSE22255), and they had a preferable performance as a diagnostic marker of the IS (the AUC of PTGS2 was 0.698; the AUC of FOS was 0.728). On the other side, the decided expression of RYR1 in samples from IS individuals was obviously lower than that of controls (GSE22255). Similarly, its ROC curve emerged well (the AUC of RYR1 was 0.703). After deeply calculation of the IS validation set (GSE16561), we were surprised to find that the expression of these DEOSG was consistent with the previous trend. Additionally, we spotted a dramatic relationship between 3 DEOSGs and immune cell infiltration, suggesting that PTGS2, FOS and RYR1 were mainly associated with Mast cells activated, Neutrophils and Plasma cells, respectively.

Recently, emerging evidence has revealed some novel programmed cell death pathways participated in the progression of IS, including ferroptosis [37]. This special cell death form is obviously featured with GSH depletion as well as MDA and ROS accumulation [38]. Ferroptosis also has been identified to cause substantial damage to the structural and functional integrity of brain tissue [39, 40]. OS and ferroptosis both play fatal roles in the complicated pathological progress in IS, however, there are still few studies on the combination of them in IS. In this work, we intersected the 3 diagnostic DEOSGs with eight upregulated ferroptosis-related DEGs and finally obtained the only overlapping 1 DEOSGs (PTGS2). Chen et al., has identified that PTGS2, as potential diagnostic biomarkers for IS, providing vital evidence about the function of ferroptosis in IS [41]. PTGS2 was also identified to play irreplaceable role in IS [42]. In this study, we further successfully constructed IS animal model (MCAO/R), after collecting brain cortical tissue of rats, RT-qPCR, western blot and IHC were all applied to verify our previous bioinformatics-based predictions. All of the three experimental results (PTGS2 was significantly increased in IS) were obviously consistent with the previous analyses. Moreover, functional experiments were executed using sh-PTGS2 transfection, revealing that the volume of cerebral infarction, neurological injure and water content were inhibited following the interference of PTGS2 compared with the MCAO group. Silencing PTGS2 can promote the better outcome of IS. More importantly, PTGS2 inhibition hindered OGD/R-induced ROS and MDA accumulation, and restores the GSH content. In addition, silencing of PTGS2 significantly eliminated the occurrence of ferroptosis triggered by OGD/R, manifested as an increase in key protein SLC7A11 and GPX4 of ferroptosis signal pathway.

Nowadays, with the development of medical technology, molecular target diagnosis and therapies play an increasing role. In our study, we injected sh-PTGS2 into rats, finding that sh-PTGS2 group could reduce the volume of cerebral infarction. Moreover, the neurological deficit scores and edema volume of rats were decreased. And sh-PTGS2 rescued the neuronal damage. Above all, silencing of PTGS2 could reduce the volume of cerebral infarction and rescue the neuronal damage. In clinical applications, early intervention and inhibition of PTGS2 may have certain therapeutic significance for reducing the area of cerebral infarction and neurological damage in IS patients. This study still has some shortcomings. The specific mechanism and pathway of PTGS2 still needs to be further explored, which is also the direction of our next research.

## CONCLUSION

Our results provided novel targets for predicting IS progression and confirmed that PTGS2, FOS and RYR1 have good diagnostic value for IS by a comprehensive bioinformatics analysis with three machine learning algorithms. Through *in vivo* and *in vitro* experimental validation, we found that the injury mechanism of IS involved oxidative stress and ferroptosis, inhibition of PTGS2 may alleviate the cerebral ischemic damage partly by anti-oxidative stress and ferroptosis. However, further meticulous works are warranted to deeply clarify the underlying mechanisms. Our findings might be conducive to clarify the pathogenesis of IS from a new perspective and provide a new horizon for clinical treatment.

## Supplementary Materials

Supplementary Table 1

## References

[1] GBD 2016 Stroke Collaborators. Global, regional, and national burden of stroke, 1990-2016: a systematic analysis for the Global Burden of Disease Study 2016. Lancet Neurol. 2019; 18:439–58. 10.1016/S1474-4422(19)30034-1 30871944 PMC6494974

[2] Stinear CM, Lang CE, Zeiler S, Byblow WD. Advances and challenges in stroke rehabilitation. Lancet Neurol. 2020; 19:348–60. 10.1016/S1474-4422(19)30415-6 32004440

[3] Mendelson SJ, Prabhakaran S. Diagnosis and Management of Transient Ischemic Attack and Acute Ischemic Stroke: A Review. JAMA. 2021; 325:1088–98. 10.1001/jama.2020.26867 33724327

[4] Kahles T, Brandes RP. NADPH oxidases as therapeutic targets in ischemic stroke. Cell Mol Life Sci. 2012; 69:2345–63. 10.1007/s00018-012-1011-8 22618244 PMC11114534

[5] Chen H, Yoshioka H, Kim GS, Jung JE, Okami N, Sakata H, Maier CM, Narasimhan P, Goeders CE, Chan PH. Oxidative stress in ischemic brain damage: mechanisms of cell death and potential molecular targets for neuroprotection. Antioxid Redox Signal. 2011; 14:1505–17. 10.1089/ars.2010.3576 20812869 PMC3061196

[6] Su XT, Wang L, Ma SM, Cao Y, Yang NN, Lin LL, Fisher M, Yang JW, Liu CZ. Mechanisms of Acupuncture in the Regulation of Oxidative Stress in Treating Ischemic Stroke. Oxid Med Cell Longev. 2020; 2020:7875396. 10.1155/2020/7875396 33178387 PMC7644298

[7] Orellana-Urzúa S, Rojas I, Líbano L, Rodrigo R. Pathophysiology of Ischemic Stroke: Role of Oxidative Stress. Curr Pharm Des. 2020; 26:4246–60. 10.2174/1381612826666200708133912 32640953

[8] Li HQ, Xia SN, Xu SY, Liu PY, Gu Y, Bao XY, Xu Y, Cao X. γ-Glutamylcysteine Alleviates Ischemic Stroke-Induced Neuronal Apoptosis by Inhibiting ROS-Mediated Endoplasmic Reticulum Stress. Oxid Med Cell Longev. 2021; 2021:2961079. 10.1155/2021/2961079 34824669 PMC8610689

[9] Cadenas S. ROS and redox signaling in myocardial ischemia-reperfusion injury and cardioprotection. Free Radic Biol Med. 2018; 117:76–89. 10.1016/j.freeradbiomed.2018.01.024 29373843

[10] Duan J, Gao S, Tu S, Lenahan C, Shao A, Sheng J. Pathophysiology and Therapeutic Potential of NADPH Oxidases in Ischemic Stroke-Induced Oxidative Stress. Oxid Med Cell Longev. 2021; 2021:6631805. 10.1155/2021/6631805 33777315 PMC7969100

[11] Qiao N, An Z, Fu Z, Chen X, Tong Q, Zhang Y, Ren H. Kinsenoside alleviates oxidative stress-induced blood-brain barrier dysfunction via promoting Nrf2/HO-1 pathway in ischemic stroke. Eur J Pharmacol. 2023; 949:175717. 10.1016/j.ejphar.2023.175717 37054938

[12] Allen CL, Bayraktutan U. Oxidative stress and its role in the pathogenesis of ischaemic stroke. Int J Stroke. 2009; 4:461–70. 10.1111/j.1747-4949.2009.00387.x 19930058

[13] Lochhead JJ, McCaffrey G, Quigley CE, Finch J, DeMarco KM, Nametz N, Davis TP. Oxidative stress increases blood-brain barrier permeability and induces alterations in occludin during hypoxia-reoxygenation. J Cereb Blood Flow Metab. 2010; 30:1625–36. 10.1038/jcbfm.2010.29 20234382 PMC2949263

[14] Gómez-Valenzuela F, Escobar E, Pérez-Tomás R, Montecinos VP. The Inflammatory Profile of the Tumor Microenvironment, Orchestrated by Cyclooxygenase-2, Promotes Epithelial-Mesenchymal Transition. Front Oncol. 2021; 11:686792. 10.3389/fonc.2021.686792 34178680 PMC8222670

[15] Asselah T, Bièche I, Laurendeau I, Martinot-Peignoux M, Paradis V, Vidaud D, Valla DC, Bedossa P, Marcellin P, Vidaud M. Significant gene expression differences in histologically "Normal" liver biopsies: Implications for control tissue. Hepatology. 2008; 48:953–62. 10.1002/hep.22411 18726958 PMC2816363

[16] Krug T, Gabriel JP, Taipa R, Fonseca BV, Domingues-Montanari S, Fernandez-Cadenas I, Manso H, Gouveia LO, Sobral J, Albergaria I, Gaspar G, Jiménez-Conde J, Rabionet R, et al. TTC7B emerges as a novel risk factor for ischemic stroke through the convergence of several genome-wide approaches. J Cereb Blood Flow Metab. 2012; 32:1061–72. 10.1038/jcbfm.2012.24 22453632 PMC3367223

[17] Barr TL, Conley Y, Ding J, Dillman A, Warach S, Singleton A, Matarin M. Genomic biomarkers and cellular pathways of ischemic stroke by RNA gene expression profiling. Neurology. 2010; 75:1009–14. 10.1212/WNL.0b013e3181f2b37f 20837969 PMC2942033

[18] Yu G, Wang LG, Han Y, He QY. clusterProfiler: an R package for comparing biological themes among gene clusters. OMICS. 2012; 16:284–7. 10.1089/omi.2011.0118 22455463 PMC3339379

[19] Zhang M, Zhu K, Pu H, Wang Z, Zhao H, Zhang J, Wang Y. An Immune-Related Signature Predicts Survival in Patients With Lung Adenocarcinoma. Front Oncol. 2019; 9:1314. 10.3389/fonc.2019.01314 31921619 PMC6914845

[20] Xv Y, Lv F, Guo H, Zhou X, Tan H, Xiao M, Zheng Y. Machine learning-based CT radiomics approach for predicting WHO/ISUP nuclear grade of clear cell renal cell carcinoma: an exploratory and comparative study. Insights Imaging. 2021; 12:170. 10.1186/s13244-021-01107-1 34800179 PMC8605949

[21] Liu Y, Cui S, Sun J, Yan X, Han D. Identification of Potential Biomarkers for Psoriasis by DNA Methylation and Gene Expression Datasets. Front Genet. 2021; 12:722803. 10.3389/fgene.2021.722803 34512732 PMC8427602

[22] Fluri F, Schuhmann MK, Kleinschnitz C. Animal models of ischemic stroke and their application in clinical research. Drug Des Devel Ther. 2015; 9:3445–54. 10.2147/DDDT.S56071 26170628 PMC4494187

[23] Xie X, Peng L, Zhu J, Zhou Y, Li L, Chen Y, Yu S, Zhao Y. miR-145-5p/Nurr1/TNF-α Signaling-Induced Microglia Activation Regulates Neuron Injury of Acute Cerebral Ischemic/Reperfusion in Rats. Front Mol Neurosci. 2017; 10:383. 10.3389/fnmol.2017.00383 29209166 PMC5702297

[24] Longa EZ, Weinstein PR, Carlson S, Cummins R. Reversible middle cerebral artery occlusion without craniectomy in rats. Stroke. 1989; 20:84–91. 10.1161/01.str.20.1.84 2643202

[25] Peng L, Zhou Y, Jiang N, Wang T, Zhu J, Chen Y, Li L, Zhang J, Yu S, Zhao Y. DJ-1 exerts anti-inflammatory effects and regulates NLRX1-TRAF6 via SHP-1 in stroke. J Neuroinflammation. 2020; 17:81. 10.1186/s12974-020-01764-x 32151250 PMC7061472

[26] Jacobs PJ, Hart DW, Bennett NC. Plasma oxidative stress in reproduction of two eusocial African mole-rat species, the naked mole-rat and the Damaraland mole-rat. Front Zool. 2021; 18:45. 10.1186/s12983-021-00430-z 34535150 PMC8447654

[27] Datta A, Sarmah D, Mounica L, Kaur H, Kesharwani R, Verma G, Veeresh P, Kotian V, Kalia K, Borah A, Wang X, Dave KR, Yavagal DR, Bhattacharya P. Cell Death Pathways in Ischemic Stroke and Targeted Pharmacotherapy. Transl Stroke Res. 2020; 11:1185–202. 10.1007/s12975-020-00806-z 32219729

[28] Zhou Y, Zhou H, Hua L, Hou C, Jia Q, Chen J, Zhang S, Wang Y, He S, Jia E. Verification of ferroptosis and pyroptosis and identification of PTGS2 as the hub gene in human coronary artery atherosclerosis. Free Radic Biol Med. 2021; 171:55–68. 10.1016/j.freeradbiomed.2021.05.009 33974977

[29] Hurford R, Sekhar A, Hughes TAT, Muir KW. Diagnosis and management of acute ischaemic stroke. Pract Neurol. 2020; 20:304–16. 10.1136/practneurol-2020-002557 32507747 PMC7577107

[30] Cojocaru IM, Cojocaru M, Sapira V, Ionescu A. Evaluation of oxidative stress in patients with acute ischemic stroke. Rom J Intern Med. 2013; 51:97–106. 24294813

[31] Yuan J, Li L, Yang Q, Ran H, Wang J, Hu K, Pu W, Huang J, Wen L, Zhou L, Jiang Y, Xiong X, Zhang J, Zhou Z. Targeted Treatment of Ischemic Stroke by Bioactive Nanoparticle-Derived Reactive Oxygen Species Responsive and Inflammation-Resolving Nanotherapies. ACS Nano. 2021; 15:16076–94. 10.1021/acsnano.1c04753 34606239

[32] Tian Y, Su Y, Ye Q, Chen L, Yuan F, Wang Z. Silencing of TXNIP Alleviated Oxidative Stress Injury by Regulating MAPK-Nrf2 Axis in Ischemic Stroke. Neurochem Res. 2020; 45:428–36. 10.1007/s11064-019-02933-y 31858374

[33] Xie W, Zhu T, Dong X, Nan F, Meng X, Zhou P, Sun G, Sun X. HMGB1-triggered inflammation inhibition of notoginseng leaf triterpenes against cerebral ischemia and reperfusion injury via MAPK and NF-κB signaling pathways. Biomolecules. 2019; 9:512. 10.3390/biom9100512 31547018 PMC6843331

[34] Imajo M, Tsuchiya Y, Nishida E. Regulatory mechanisms and functions of MAP kinase signaling pathways. IUBMB Life. 2006; 58:312–7. 10.1080/15216540600746393 16754324

[35] Zhou YY, Li Y, Jiang WQ, Zhou LF. MAPK/JNK signalling: a potential autophagy regulation pathway. Biosci Rep. 2015; 35:e00199. 10.1042/BSR20140141 26182361 PMC4613668

[36] Slattery ML, Mullany LE, Sakoda LC, Wolff RK, Samowitz WS, Herrick JS. The MAPK-Signaling Pathway in Colorectal Cancer: Dysregulated Genes and Their Association With MicroRNAs. Cancer Inform. 2018; 17:1176935118766522. 10.1177/1176935118766522 29636593 PMC5888819

[37] Yang WS, Stockwell BR. Ferroptosis: Death by Lipid Peroxidation. Trends Cell Biol. 2016; 26:165–76. 10.1016/j.tcb.2015.10.014 26653790 PMC4764384

[38] Jiang X, Stockwell BR, Conrad M. Ferroptosis: mechanisms, biology and role in disease. Nat Rev Mol Cell Biol. 2021; 22:266–82. 10.1038/s41580-020-00324-8 33495651 PMC8142022

[39] Xu Y, Li K, Zhao Y, Zhou L, Liu Y, Zhao J. Role of Ferroptosis in Stroke. Cell Mol Neurobiol. 2023; 43:205–22. 10.1007/s10571-022-01196-6 35102454 PMC11415219

[40] Yu Y, Yan Y, Niu F, Wang Y, Chen X, Su G, Liu Y, Zhao X, Qian L, Liu P, Xiong Y. Ferroptosis: a cell death connecting oxidative stress, inflammation and cardiovascular diseases. Cell Death Discov. 2021; 7:193. 10.1038/s41420-021-00579-w 34312370 PMC8313570

[41] Chen G, Li L, Tao H. Bioinformatics Identification of Ferroptosis-Related Biomarkers and Therapeutic Compounds in Ischemic Stroke. Front Neurol. 2021; 12:745240. 10.3389/fneur.2021.745240 34707562 PMC8542983

[42] Lin W, Wang Y, Chen Y, Wang Q, Gu Z, Zhu Y. Role of Calcium Signaling Pathway-Related Gene Regulatory Networks in Ischemic Stroke Based on Multiple WGCNA and Single-Cell Analysis. Oxid Med Cell Longev. 2021; 2021:8060477. 10.1155/2021/8060477 34987704 PMC8720592

